# Trust, Anxious Attachment, and Conversational AI Adoption Intentions in Digital Counseling: A Preliminary Cross-Sectional Questionnaire Study

**DOI:** 10.2196/68960

**Published:** 2025-04-22

**Authors:** Xiaoli Wu, Kongmeng Liew, Martin J Dorahy

**Affiliations:** 1 School of Psychology, Speech and Hearing University of Canterbury Christchurch New Zealand

**Keywords:** attachment style, conversational artificial intelligence, CAI, perceived trust, adoption intentions, CAI counseling, mobile phone

## Abstract

**Background:**

Conversational artificial intelligence (CAI) is increasingly used in various counseling settings to deliver psychotherapy, provide psychoeducational content, and offer support like companionship or emotional aid. Research has shown that CAI has the potential to effectively address mental health issues when its associated risks are handled with great caution. It can provide mental health support to a wider population than conventional face-to-face therapy, and at a faster response rate and more affordable cost. Despite CAI’s many advantages in mental health support, potential users may differ in their willingness to adopt and engage with CAI to support their own mental health.

**Objective:**

This study focused specifically on dispositional trust in AI and attachment styles, and examined how they are associated with individuals’ intentions to adopt CAI for mental health support.

**Methods:**

A cross-sectional survey of 239 American adults was conducted. Participants were first assessed on their attachment style, then presented with a vignette about CAI use, after which their dispositional trust and subsequent adoption intentions toward CAI counseling were surveyed. Participants had not previously used CAI for digital counseling for mental health support.

**Results:**

Dispositional trust in artificial intelligence emerged as a critical predictor of CAI adoption intentions (*P*<.001), while attachment anxiety (*P=*.04), rather than avoidance (*P*=.09), was found to be positively associated with the intention to adopt CAI counseling after controlling for age and gender.

**Conclusions:**

These findings indicated higher dispositional trust might lead to stronger adoption intention, and higher attachment anxiety might also be associated with greater CAI counseling adoption. Further research into users’ attachment styles and dispositional trust is needed to understand individual differences in CAI counseling adoption for enhancing the safety and effectiveness of CAI-driven counseling services and tailoring interventions.

**Trial Registration:**

Open Science Framework; https://osf.io/c2xqd

## Introduction

### Conversational Artificial Intelligence in Mental Health

Conversational artificial intelligence (CAI) has rapidly captured global attention since its emergence in recent years. It has permeated various facets of human life and continues to attract a growing user base worldwide due to its unparalleled impact on the way people access knowledge, present ideas, and interact. Commercially available CAIs, including Replika (developed by Luka Inc, Replika is a chatbot designed to be a conversational agent and personal companion, using artificial intelligence (AI) to simulate human-like conversations; its primary purpose is to provide users with an AI friend that can listen, respond empathetically, and help users reflect on their thoughts and feelings. It is often used for mental health support, companionship, and improving emotional well-being [1]) and Pi (Developed by Inflection AI, Pi is a CAI designed to provide a range of task-based features, including emotional support, learning assistance, and personalized interactions; it is specifically tailored to engage users in meaningful conversations, making it useful for various purposes, such as mental health support, learning new languages, and relationship advice), are powered by large language models (LLMs) with deep learning–based natural language processing to enable human-like voice or text interactions with users. They offer a wide range of services such as information retrieval, task completion, entertainment, and even mental health support [2]. In the context of mental health support, CAI is used in various counseling settings like delivering psychotherapy, providing psychoeducational content, and offering support such as companionship or emotional aid [3]. In this paper, we define CAIs as chatbots that use LLMs to generate naturalistic text, which is different from traditional rule-based conversational agents that operate mainly on predefined scripts, such as customer-oriented chatbots commonly used in sales and marketing.

One increasingly common usage of these anthropomorphic CAIs has been for counseling purposes in mental health settings to improve the overall quality of communications [4]. Gaffney et al [5] conducted a systematic review of 13 studies on the application of conversational agents (including CAIs) in psychotherapeutic settings and found that overall, CAIs showed promising results in terms of effectiveness and acceptability for addressing mental health issues in users. More recently, Li et al [6] conducted a meta-analysis of 15 randomized controlled trials specifically focusing on CAI counseling, and found that CAIs showed a significant decrease in depression and distress symptoms, especially when used with clinical, subclinical, and older adult populations. These findings suggest that CAI has the potential to effectively address mental health issues. Furthermore, the accessibility and user-friendly nature of CAI have also made them a promising tool for delivering mental health care to a wider population at a faster response rate and at an affordable cost, compared with traditional in-person therapies. It offers hope for overcoming long-lasting barriers, such as social stigma and the demand-supply imbalance, that weigh down traditional mental health care services [7].

Despite the benefits CAI counseling could potentially bring to mental health care, it also poses many risks and challenges, such as misleading responses, privacy infringement, and ethical concerns, to name a few [8]. For instance, counseling typically involves a high degree of self-disclosure, which in the context of CAI counseling can be problematic. Users may share sensitive and personal information that, if not properly protected, could be vulnerable to data breaches or misuse. Furthermore, the algorithms used by CAIs might not fully grasp the nuances of human emotions and mental health issues, potentially providing inappropriate or harmful responses (eg, spreading misinformation, professing their love to users, and sexually harassing minors) [9]. In addition, users of CAI counseling may be more susceptible to developing maladaptive behaviors (eg, addiction) as most counseling CAIs are designed to form social-emotional bonds with its users. While CAI therapies are intended to improve users’ psychological well-being, they also risk users developing overreliance and social withdrawal [10]. Without caution in its application and a thorough understanding in human-CAI interaction in counseling settings, the unpredictability in CAI responses could lead to adverse psychological consequences on the user.

How should we weigh the pros and cons of adopting CAI counseling for mental health support? Most of the relevant literature [2,11] acknowledges the significant potential of CAI therapies in providing therapeutic support and underscores the necessity for further exploration and implementation, but also highlights the importance of recognizing and meticulously managing the risks associated with CAI therapies through rigorous research and well-defined guidelines. Furthermore, regardless of the concerns related to the use of CAI for psychological support, there are already CAIs that provide easy access to task-oriented features designed for mental health purposes. For example, a wide range of diverse task-oriented features offered by Pi fall under this category, such as venting, self-care for anxiety, and relationship advice [12]. Particularly, the younger generation may be more open to trying new technologies, making them more vulnerable to potential harms from poorly regulated or non–evidence-based CAI therapies. Therefore, to ensure the safe and effective integration of CAI into mental health services, it is crucial to understand the factors influencing CAI adoption, including potential predictors and barriers. However, research is relatively lacking in this area [7,10]. Studies addressing the factors associated with individual adoption of CAI counseling is needed to comprehend the psychological mechanisms underpinning the formation of human-CAI relationships. This study was designed to address this gap, by examining individual differences in attachment styles and perceived trust as predictors of CAI adoption for mental health care.

Numerous studies have demonstrated attachment style to be a reliable predictor for various relational outcomes [13,14], including the relationship between humans and technology. Meanwhile, trust is considered as another key factor in the context of technology adoption and use, especially in the domain of AI adoption due to risks related to its complexity as mentioned earlier [15]. Therefore, for this study, perceived trust and attachment styles were both examined as potential pertinent variables that might account for individual differences in CAI adaption in the context of digital counseling.

### Trust as a Potential Predictor for CAI Counseling Adoption

Based on the theoretical framework developed by McKnight [16], trust is the extent to which a person has confidence in, and is ready to rely on, another entity (in this case, CAI). The formation of trust in information technology goes through different stages, each influenced by distinct factors and mechanisms. Considering CAI as a relatively recent technology, we assume that most individuals would have no previous experiences with CAI counseling. Therefore, the primary focus of this study was on the initial stage of trust building, which pertains to establishing trust with an unfamiliar party or service without previous interaction. Numerous studies have examined the individual process of technology adoption from the perspective of trust formation, where the entity being trusted is a technology such as an information system or a recommendation agent. For example, when examining the factors that influence digital voice assistant use, Fernandes and Oliveira [17] found a positive link with perceived trust. Kasilingam [18] investigated intentions toward using smartphone chatbots for purchasing decisions and found that trust positively influenced participants’ willingness to use chatbots for mobile shopping purposes. However, studies have not yet examined trust as a predictor for the adoption intention of CAI counseling for psychological support before engagement. While a recent review [19] identifies trust as a key predictor of CAI adoption in health care, including mental health care settings, the CAIs discussed in this review reflect a broader, more general definition of CAIs, including those that use prewritten scripts, which fall outside the scope of our research. Research specifically studying the relationship between trust and adoption intention of advanced CAI counseling (eg, ChatGPT [OpenAI] relying on contemporary reinforcement -learning with human feedback-based LLM technology) is still lacking. Additional research is needed to examine the replicability and reliability of these conclusions within the context of advanced CAI counseling technologies. Furthermore, given that CAI counseling for psychological support involves deeper emotional bonding and personal information, trust may play a significantly different role compared with CAI applications for other aspects of mental health care, such as diagnosis or treatment adherence. Studies examining the formation of trust on primitive, pre-LLM chatbot systems have found positive associations between perceived trust and chatbot adoption, which may generalize to explain how initially perceived trust shapes individuals’ behaviors in considering the use of CAI counseling. Hence, in this study, we tested whether perceived trust can predict CAI counseling adoption.

### Attachment Theory and Styles

Attachment theory, initially developed by John Bowlby, is a psychological framework that describes how infants learn to interact with their caregivers [20-23]. It was later expanded and adapted to explain the dynamics of both long and short-term interpersonal relationships between humans [24]. A key concept within this theory is the idea of “internal working models (IWMs),” which are shaped by early interactions with primary caregivers. The nature of these interactions, whether they are nurturing, inconsistent, or neglectful, greatly influences the types of IWMs developed. When a caregiver consistently responds to a child’s needs in a caring, supportive manner, it tends to foster a positive IWM, while inconsistent or neglectful nurturing is more likely to lead to the formation of negative IWMs. These IWMs serve as mental templates that individuals use to perceive themselves and others, and influence their attributions, perceptions, and emotional understandings of these connections. In essence, they tend to serve as a prototype to determine an individual’s expectations and behaviors in subsequent relationships [25-27].

Attachment styles are commonly presented as secure attachment, anxious attachment, avoidant attachment, and disorganized attachment. However, disorganized attachment is often viewed as the most unpredictable type due to its lack of organization in how the child (and later adult) responds to their attachment figures, characterized by push-pull dynamics that lead to inconsistent and conflicted coping strategies [28]. This variability makes it challenging to draw reliable and accurate measurements. For that reason, disorganized attachment was not examined in this study.

According to Bretherton and Munholland [29], attachment style can be understood as the manifestation of people’s underlying IWMs. The IWM of attachment avoidance is thought to manifest a positive view of self (as worthy of love and nurturance) and a negative view of others (as unresponsive and untrustworthy). Conversely, attachment anxiety is thought to be associated with an IWM that contains a negative view of self and a positive view of others. Finally, secure attachment is the combination of positive views of both self and others. Securely attached individuals are more capable of forming and maintaining close relationships, with higher commitment, intimacy, love, and satisfaction in such relationships. As for the two insecure attachment styles, avoidant attachment is defined by devaluation of the importance of close relationships, avoidance of intimacy and dependence, and decreased engagement in attachment behavior, while anxious attachment involves preoccupation with the availability and responsiveness of attached figures, fear of separation, and abandonment [24,30].

### Attachment Insecurity as a Potential Predictor for CAI Counseling Adoption

While attachment styles are typically associated with interpersonal relationships, Hodge and Gebler-Wolfe [31] found that inanimate objects, such as smartphones, could also be perceived as attachment objects for anxiously attached individuals to feel secure, and reduce unpleasant feelings such as loneliness and boredom. This illustrates how attachment theory can be used as a framework to understand a person’s relationship with technology. Beyond smartphones, Birnbaum et al [32] found that humans desire the presence of robots in stressful circumstances in a similar manner to their proximity-seeking behavior toward human attachment figures, suggesting that attachment might also play a similarly important role in human-CAI interactions. Given that CAI is a relatively nascent technology, especially in its application for mental health support, there is, to the best of our knowledge, no previous literature investigating the direct relationships between attachment styles and CAI use. The closest study explored the influence of different attachment styles on perceived trust in broadly defined AI; here Gillath et al [33] found that attachment anxiety was associated with lower trust in AI. Furthermore, participants’ trust in AI was reduced when their attachment anxiety was enhanced and increased when their attachment security was boosted. Accordingly, consistent with the positive association between perceived trust and CAI adoption, we expected to find a negative association between attachment anxiety and CAI adoption intention in our study.

Meanwhile, although Gillath et al [33] found no significant effect of attachment avoidance on trust in AI, likely due to the inhibited nature of avoidant individuals, we believe it is important and necessary to include both attachment styles in this study in order to provide comprehensive insights into an underexplored area of research. n this study, we take a broader approach by also hypothesizing the relationship between attachment avoidance and CAI adoption. Insecure attachment styles, including both anxious attachment and avoidant attachment, are generally associated with lower levels of trust. For example, a number of studies on human relations have shown that attachment security is associated with more trust, whereas attachment insecurity is associated with less trust in other humans [34-36]. It may thus be reasonable to hypothesize that higher attachment avoidance will predict lower CAI adoption intention. This hypothesis is grounded in the understanding that individuals with high attachment avoidance may be less inclined to trust, and therefore, are less likely to adopt new technologies like CAI.

### Research Hypotheses

Therefore, as a first step toward the eventual aim of promoting safer adoption and designing better CAI for mental health support, this study examined how perceived trust and attachment insecurity (ie, attachment anxiety and attachment avoidance) are associated with CAI adoption intentions. We propose the following two hypotheses:

First (hypothesis 1), due to the positive association found between the perceived trust and primitive chatbots adoption in the previous literature**,** higher trust in CAI counseling would predict higher adoption intentions for CAI counseling, after controlling for general confounding variables of age and gender.

Second (hypothesis 2), due to the association between insecure attachment styles (ie, anxiety and avoidance) and lower levels of trust, individuals with higher insecure attachments would show lower adoption intentions for CAI counseling, after controlling for age and gender.

To test the above hypotheses, a cross-sectional web-based survey was conducted. As no previous study has examined the human-CAI relationship through the perspective of attachment styles, this preliminary study may provide novel insights into this area and contribute to the existing literature on attachment and technology-mediated relationships. All hypotheses and methods were preregistered before data collection at Open Science Framework [37] and eventual deviations from the preregistration are detailed in Multimedia Appendix 1.

## Methods

### Participants

Based on an a priori power analysis, a minimum sample size of 146 was recommended to detect an effect size of *F*_2_=0.075 with 95% power and alpha at .05 using a linear multiple regression with 6 predictors. The effect size was obtained from the findings of Gritti et al [38] on the effect of avoidant attachment on social network mobile app use. A total of 274 participants (aged 18 y and older, with American nationality or residence) were initially recruited through a large and diverse participant pool from the “Prolific” platform (prolific website; Prolific is a web-based service that provides access to a diverse pool of participants [initially recruited from word-of-mouth and social media] who opt-in to participate in studies listed on the platform. Eligible participants from the Prolific platform are notified through email or their Prolific dashboard. Prolific matches studies to participants based on prescreened criteria. Notifications are presented with necessary information, such as the study title, brief description of the study, estimated time commitment, and payment details clearly displayed, etc) between December 2023 and January 2024. Furthermore, 35 participants’ entries were removed due to incomplete data or ineligibility (eg, participants with previous CAI counseling experience as we were only interested in their adoption intention before engagement) responses, leaving a final sample size of 239 participants. The gender ratio of participants was nearly balanced (Table 1). Most of the participants identified as European Americans (153/239, 64% European; 37/239, 15% Asian; 27/239, 11% African American; and 22/239, 8% Native American and others) with a wide age range from 18 to 74 (mean 36.9, SD 12.4) years. For a breakdown of participant demographics, refer to Table 1.

**Table 1:** Demographic data breakdown for all participants (N=239).

Variables		Frequency, n (%)
**Gender**		
	Women	114 (47.7)
	Men	119 (49.8)
	Other	6 (2.5)
**Ethnicity**		
	European (Caucasian)	153 (64)
	Asian	37 (15.5)
	Black or African American	27 (11.3)
	American Indian or Alaska Native	3 (1.3)
	Other	17 (7.1)
	Native Hawaiian or other Pacific Islander	1 (0.4)
	Prefer not to say	1 (0.4)
**Education level**		
	High school or equivalent	29 (12.1)
	College or associated degree	63 (26.4)
	Bachelor’s degree	90 (37.7)
	Postgraduate degrees	55 (23)
	Others	2 (0.8)
**PEDT^a^**		
	Negative	8 (3.4)
	Neutral	49 (20.5)
	Positive	182 (76.2)
**Familiarity with CAI^b^ counseling**		
	Not familiar at all	116 (48.5)
	Slightly familiar	81 (33.9)
	Moderately familiar	31 (13)
	Very familiar	10 (4.2)
	Extremely familiar	1 (0.4)

^a^PEDT: previous experience with digital technologies.

^b^CAI: conversational artificial intelligence.

### Materials and Procedure

#### Overview

After reading the information sheet and providing consent to participate, participants proceeded to a survey consisting of multiple blocks in a predetermined order (ie, attachment styles, trust toward CAI counseling, intention of use for CAI counseling, and demographic questions). The item order in each scale was randomized to reduce response bias, and an attention check question was included in the survey.

#### Adult Attachment Style

Adult attachment style was measured using the close relationship version of Revised Adult Attachment scale [39]. This scale contains 2 subscales with 6 items assessing anxious attachment (eg, “I often worry that other people don’t really love me,” Cronbach α=0.91) and the other 12 items measuring avoidant attachment (eg, “I find it difficult to allow myself to depend on others,” Cronbach α=0.88). Participants were asked to think about their close relationships with people important to them, such as family members, romantic partners, and close friends, and to rate each statement on a 5-point Likert scale, ranging from 1 (strongly disagree) to 5 (strongly agree). Anxious attachment scores and avoidant attachment scores were computed by taking the average of items within each subscale, with certain items being reverse-scored.

#### Trust in CAI Counseling

The concept of CAI counseling is still relatively new to most people. In order to introduce its applications in mental health support, we adapted a news article illustrating the use of CAI in these contexts (Multimedia Appendix 1) for participants to read before completing the survey questions on trust in CAI in the setting of mental health support. This was edited to be as neutral in tone as possible and to remove references to gendered pronouns. A 12-item human-computer trust scale was adapted from previous research [40] (eg, “I think that CAI is competent and effective in providing mental support,” Cronbach α=0.94) with each statement rated on a 5-point Likert scale, ranging from 1 (strongly disagree) to 5 (strongly agree). Scores for trust were calculated by averaging the items after reverse-scoring the relevant items.

The vignette plays a crucial role in this study, as it provides a contextual scenario to introduce and illustrate the practical application of CAI in mental health care. Given that CAI is still an emerging technology, particularly in the context of mental health support, the vignette helps bridge potential gaps in participants’ understanding. This was especially important in case randomly enrolled participants were unfamiliar with CAI counseling or had never encountered it before.

#### CAI Counseling Adoption Intentions

CAI adoption intentions for mental health support were measured with a single-item measure, “How likely are you to try a counseling service based on CAI for mental health support in future (if needed)?” using a 5-point Likert-type scale (1=extremely unlikely, 5=extremely likely).

#### Demographics

Participants were asked to answer demographic questions on their age, gender, and education level. In addition, participants were asked about their previous experience with digital technology on a single-item measure, “How is your previous experience with digital technology in general?” (negative, neutral, or positive), and their familiarity with CAI’s counseling function for mental health support on another single-item measure, “How familiar are you with the counseling function of CAI?” A 5-point Likert scale from 1 (not familiar at all) to 5 (extremely familiar) was adopted. In addition, previous CAI counseling experience was assessed on a single question, “Have you used CAI for mental health support before? If yes, please tell us more about your experience with it (eg, usability, effectiveness, satisfaction, and motivators for first engagement with CAI, etc.) if you would like to share.”

### Ethical Considerations

This study was approved by the Human Research Ethics Committee at the University of Canterbury, HREC 2023-120-LR. Participants received compensation of GBP 1.00 (US $1.28) for completing the survey.

All participants were required to carefully read the information sheet, which included essential details such as the research purpose, participation procedure, anonymity assurance, and potential benefits of participation. Participants were informed they could withdraw from the survey at any point. Completion and submission of the survey indicated participants’ consent to participate.

## Results

### Demographics and Descriptive Statistics

Table 1 presents a breakdown of participant demographics. Note that nearly half the participants reported a lack of familiarity with the counseling aspects of CAI.

Descriptive statistics and a correlation matrix among all variables of interest are illustrated in Table 2. Attachment anxiety and avoidance were significantly and positively correlated with each other, which supports the dimensional rather than categorical nature of attachment styles. Furthermore, there was a negative and significant association between age and anxious attachment. Older participants tend to have lower anxiety associated with attachment. A strong significant correlation was found between trust and CAI adoption; higher trust was linked with greater intention of using CAI for mental health support.

**Table 2:** Descriptive statistics correlations matrix for all variables of interest.

Variable	Mean (SD)	A-anxiety^a^	A-avoidance^b^	Trust	CAI^c^ adoption
A-anxiety	2.95 (1.11)				
A-avoidance	2.95 (0.78)	.67^d^			
Trust	2.83 (0.91)	–.02	–.10		
CAI adoption	2.80 (1.38)	.06	–.04	.77^e^	
Age	36.93 (12.36)	–.24^e^	–.10	.05	.07

^a^A-anxiety: attachment anxiety.

^b^A-avoidance: attachment avoidance.

^c^CAI: conversational artificial intelligence.

^d^*P<*.01.

^e^*P*<.001.

### Confirmatory Results

To test the first hypothesis, a hierarchical ordinal logistic regression was conducted to examine the relationship between perceived trust in CAI counseling and CAI adoption intention, given that the outcome variable (CAI adoption intention) was a single-item categorical variable. Table 3 reports the full breakdown of results for each model (using standardized regression coefficients for all predictors). For the first step, age and gender were entered into the model. This step aimed to control for these demographic variables’ effects on the outcome variable. Subsequently, the variable of interest—perceived trust—was added into the model to see whether the perceived trust explained significant variance in participants’ CAI adoption intention above and beyond the effect of age and gender.

**Figure 1:**
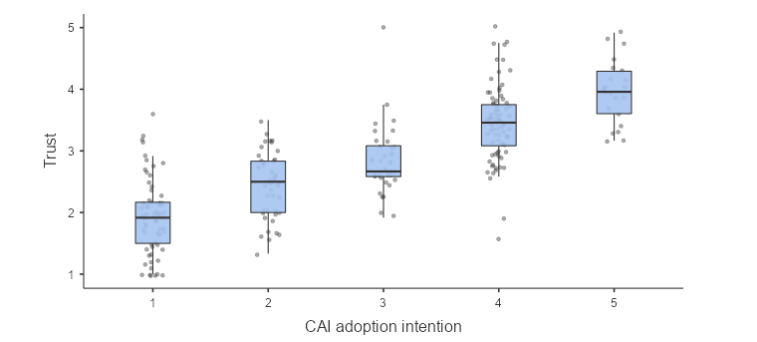
Descriptive box plot illustrates the relationship between perceived trust and CAI adoption intention. CAI: conversational artificial intelligence.

Aligning with hypothesis 1, perceived trust in CAI counseling emerged as a strong predictor of CAI adoption intention (*b*=2.62, 95% CI 2.19-3.09, *P*<.001, odds ratio [OR] 13.70). This suggests the higher the trust levels participants have toward CAI’s counseling, the more willing they are to use CAI for mental health support, after controlling for age and genders. This tendency is also apparent in the box plot in which perceived trust was plotted against CAI adoption intention in Figure 1.

Considering our aim of examining adoption in initial stages of trust-building with counseling CAI, we conducted a robustness check by repeating the analysis with the subset of participants who reported “not familiar at all” with counseling CAI (n=116). For this sample, perceived trust in CAI counseling was still a strong predictor of CAI adoption intention (*b*=2.72, 95% CI 2.07-3.45, *P*<.001, OR 15.10), after controlling for age and gender.

To test the second hypothesis, we repeated the hierarchical ordinal logistic regression analysis to see if attachment insecurity predicted CAI adoption intention. Age and gender were included in the first step, followed by anxious attachment and avoidant attachment scores as the second step for predicting CAI adoption intention. Full results can be found in Table 4.

**Figure 2:**
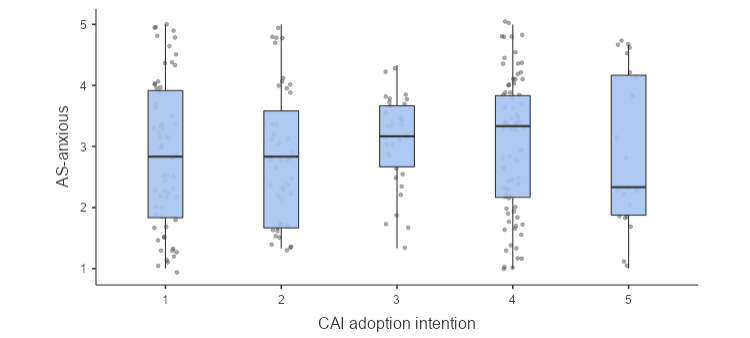
Descriptive box plot illustrating the relationship between attachment anxiety and conversational artificial intelligence adoption intention. AS-anxious: anxious attachment style; CAI: conversational artificial intelligence.

In contrast with hypothesis 2, we observed a small but positive significant effect of attachment anxiety on CAI adoption intention when age and gender were controlled (*b*=0.33, 95% CI 0.02-0.64, *P*=.04, OR 1.39). This means people with higher attachment anxiety are more likely to adopt CAI for mental support. It is contrary to the direction (ie, negative) that was theorized in hypothesis 2. However, this effect did not appear to be robust, as it was not significant in a zero-order correlation (Table 2). As shown in Figure 2, there was no clear pattern between attachment anxiety and CAI adoption intention before controlling for age and gender. No other significant relationships were found including between attachment avoidance and CAI adoption intentions.

To align with our aim of examining adoption in initial stages of trust-building with counseling CAI, we conducted a robustness check by repeating the analysis with the subset of participants who reported “not familiar at all” with counseling CAI (n=116). For this sample, attachment anxiety significantly predicted of CAI adoption intention (*b*=0.55, 95% CI 0.09-1.02, *P*=.02, OR 1.74), but not attachment avoidance (*b*=–0.54, 95% CI –1.148 to 0.064, *P*=.08, OR 0.59), after controlling for age and gender.

**Table 3:** Regression coefficients for conversational artificial intelligence adoption as a function of multiple variables (N=239).

Predictor variables		Step 1, *b* (95% CI)		SE	*P* value	Step 2, *b* (95% CI)	SE	*P* value
Age		0.01(–0.01 to 0.03)		0.01	.26	0.001 (–0.02 to 0.02)	0.01	.92
**Gender**								
	Men-Women		0.24 (–0.23 to 0.70)	0.24	.32	–0.35 (–0.88 to 0.17)	0.27	.19
	Other-Women		–0.73 (–2.19 to 0.65)	0.71	.30	–0.49 (–2.26 to 1.16)	0.86	.57
Trust in CAI^a^ Counseling		—^b^		—	—	2.62 (2.19 to 3.09)	0.23	<.001
R^2^_McF_^c^		0.01		—	—	0.29	—	—
R^2^_McF_ change		—		—	—	0.28	—	—

^a^CAI: conversational artificial intelligence*.*

^b^Not applicable.

^c^R^2^_McF_: McFadden’s R-squared.

**Table 4:** Regression coefficients for conversational artificial intelligence adoption as a function of multiple variables (N=239).

Predicting variables		Step 1, *b* (95% CI)	SE	*P* value	Step 2 *b* (95% CI)	SE	*P* value
Age		0.01 (–0.01 to 0.03)	0.01	.26	0.02 (–0.004 to 0.04)	0.01	.12
**Gender**							
	Men-Women	0.24 (–0.23 to 0.70)	0.24	.32	0.28 (–0.19 to 0.75)	0.24	.24
	Other-Women	–0.73 (–2.19 to 0.65)	0.71	.30	–0.64 (–2.12 to 0.76)	0.72	.37
Attachment anxiety		—^a^	—	—	0.33^b^ (0.02 to 0.64)	0.16	.04
Attachment avoidance		—	—	—	–0.36 (–0.77 to 0.06)	0.21	.09
R^2^_McF_^c^		.005	—	—	.012	—	—
R^2^_McF_ change		—	—	—	.007	—	—

^a^Not applicable.

^b^*P≤*.05.

^c^R^2^_McF_: McFadden’s R-squared.

## Discussion

### Principal Findings

In this study, perceived trust and attachment insecurity (ie, attachment anxiety and attachment avoidance) were examined as factors that influence the dependent variable—CAI adoption intention. In hypothesis 1, we assumed that higher trust in CAI counseling would be associated with a stronger adoption intention, with age and gender controlled for their effects. The results supported this hypothesis, as trust appeared to be a strong predictor of participants’ intention to use CAI for mental health support. In addition, in hypothesis 2, anxious attachment and avoidant attachment were proposed to be negatively linked to CAI adoption intention, after controlling for age and gender. Surprisingly, the results did not support this hypothesis. Specifically, avoidant attachment was not a significant predictor of CAI adoption intention, while anxious attachment was found to be a significant predictor with a small effect, but only after controlling for age and gender. Contrary to our original expectation, a greater level of attachment anxiety was found to predict a stronger CAI adoption intention.

### Implication of Primary Findings

When it comes to the implementation of a novel but uncertain emerging technology like CAI, it is important to understand users’ psychology and resultant behaviors at different stages of interaction, to understand how to achieve safe relationships and positive, effective outcomes. There is a critical distinction in the focus between the pre-engagement stage, such as individual users' intentions to adopt the technology, and the post -engagement stage, such as usage patterns and addiction. As CAI for mental health support has not achieved widespread usage, we focused on the pre-engagement stage in order to examine and describe potential predictors that drive individual engagement with CAI in the context of mental health support.

To our knowledge, this is the first study looking at the relationship between trust and CAI adoption intention for the specific purpose of mental health support. These findings are highly important as they underscore the critical role of trust in the adoption of CAI for mental health support. Given the sensitive nature of mental health, establishing and enhancing trust in CAI systems is paramount. Although many people may not yet be familiar with the potential of CAI in providing mental health support, this may change as CAI becomes more widely accepted and integrated into various fields. In times of urgent need, when human resources are unavailable or delayed, CAI could emerge as a valuable and appealing option for those seeking mental health support, prompting them to explore its potential for engagement. Therefore, user safety, wider acceptance, and use of this technology—all call for developers to prioritize robust security protocols and transparent privacy policies to enhance users’ trust, including clear communication about how data are collected, stored, and used. Meanwhile, establishing and adhering to ethical standards is essential. This includes ensuring the AI’s recommendations are safe, accurate, and unbiased. Providing users with training and resources to understand how CAI systems work can also demystify the technology and build trust.

Future research should focus on identifying specific factors that build or hinder trust in CAI, particularly in diverse populations, and explore interventions that could mitigate trust-related barriers. In addition, it will be crucial to investigate how trust interacts with other psychological variables, such as attachment styles, to fully understand its role in CAI adoption. Notably, a relatively small effect of attachment anxiety on CAI adoption intention was detected after controlling for age and gender. One possible explanation for the observed effect could be that lower levels of attachment anxiety among older individuals diluted the overall impact of attachment anxiety on adoption intention. Recent research has indicated age as an effective demographic factor to predict AI adoption. For example, Shandilya and Fan [41] found that older adults are less likely to use AI products than younger generations. Similarly, Draxler and colleagues [42] found that early adopters of LLMs, such as ChatGPT, tended not to include individuals from relatively older age groups. This calls for further research incorporating theoretical frameworks and broader contextual and demographic variables to clarify the roles of age and gender in CAI adoption, particularly in the context of counseling therapies for mental health.

Furthermore, to our surprise, higher attachment anxiety was linked with higher adoption intention. One explanation for this unexpected positive association between attachment anxiety and CAI adoption intention might be the constant and excessive need for validation, reassurance, and emotional support which characterizes anxiously attached individuals [43]. Unlike individuals with attachment avoidance, who tend to suppress or ignore their attachment needs to avoid the discomfort caused by fear of abandonment, those with anxious attachment cope by seeking additional attention and affirmation to alleviate their fears and insecurities. Due to the anthropomorphic, nonjudgmental, constantly accessible, and responsive nature of CAI counseling, anxiously attached individuals might consider CAI as a potential attachment object as well as a secure base, for comfort and reassurance seeking whenever needed. This reassurance-seeking behavioral pattern demonstrated by anxiously attached people was also observed in studies on attachment toward inanimate or nonhuman objects and entities, such as smartphones [31] and robots [32]. On the other hand, attachment anxiety is a key indicator of insecure attachment, with individuals exhibiting lower levels of attachment anxiety generally being more securely attached. This higher sense of security may foster greater confidence in their interpersonal skills, making them more comfortable seeking assistance or support from other individuals, as well as in communicating negative or challenging emotions to others. These may also reduce their need for CAI counseling.

Our findings indicate that CAI could be particularly attractive and beneficial for anxiously attached individuals, potentially filling gaps where traditional support is inaccessible or unavailable. Compared with those with attachment avoidance, individuals with attachment anxiety may be more likely to engage with CAI for psychological support, potentially becoming a key demographic within its user base. CAI systems could benefit from tailoring their communication styles to address the unique needs of users with attachment anxiety, ensuring these technologies provide desired emotional support and safe engagement.

While recognizing the significant potential of CAI for psychological support, we believe it is also equally crucial to be aware of the associated risks that might arise in human-CAI interaction. Research has consistently linked attachment anxiety with increased social media use and addiction [44-47]. Consequently, individuals with attachment anxiety may also be more susceptible to developing unhealthy dependencies on CAI in the postengagement phase. Proactively identifying solutions and applying appropriate strategies during the design phase can mitigate potential negative outcomes. It is essential to alert CAI designers to potential maladaptive behaviors associated with CAI use. Integrating protective measures, such as timely advice and interventions, can help safeguard the user experience and optimize therapeutic outcomes, particularly for users with attachment anxiety.

In terms of attachment avoidance, the lack of a significant result is congruent with previous research [48,49]; avoidant-attached individuals have a need to deactivate the attachment system (eg, by inhibiting proximity-seeking behaviors), and this tendency often makes it difficult to observe and capture their avoidant nature in surveys. To be more specific, individuals with attachment avoidance often prefer self-reliance and independence. They are more likely to maintain emotional distance to feel safe rather than seek emotional support, which might lead to a weaker or nonexistent link between attachment avoidance and CAI adoption intention, similar to the results found in our study.

To the best of our knowledge, this study is the first to explore the relationship between attachment styles and CAI adoption in the context of CAI-based therapies. More evidence is needed to determine whether our current findings (in both significance and direction) are replicable and reliable. If attachment styles, particularly attachment anxiety, prove to be a consistent predictor of CAI adoption intention, this could inform the development of more customized designs that promote safer interactions and outcomes that are more effective.

### Trust in Generalized AI and CAI

In addition, past studies [33] have already established a relationship between attachment styles and trust in generalized AI. However, our results suggest that this may not necessarily replicate in the context of CAI. According to the results of correlation matrix illustrated in Table 2, both attachment anxiety and avoidance were not significantly correlated with trust in CAI counseling in our study. Therefore, trust was not assessed as a mediator between attachment anxiety and CAI adoption intentions. Specifically, perceived trust was not significantly associated with attachment anxiety (β=.091, *P*=.30) and attachment avoidance (β=–.161, *P*=.07). This finding is inconsistent with the conclusion (ie, higher attachment anxiety predicts lower trust) found by Gillath et al [33] in their study, that we previously relied on in hypothesizing a negative direction between attachment anxiety and CAI adoption intention in hypothesis 2. Hence, this inconsistency could signify a more complex relationship between attachment styles and perceived trust in CAI adoption.

To contextualize these results in understanding this inconsistency, one possible explanation could be that participants’ attachment systems may not have been sufficiently activated in this study. According to a review conducted by Campbell and Marshall [50], attachment theory is interactionist in nature, particularly attachment anxiety. Highly anxious individuals may exhibit heightened distress responses when they perceive cues as threats to their relationships. However, in the absence of such cues or when their security needs are fulfilled, they often show similar proximity-seeking tendencies in affect, cognition, and relationship processes to people with low anxiety levels. This suggests that when the attachment system is not effectively activated, it could potentially lead to weaker or contradictory associations between attachment styles and attachment-related behaviors, such as the relationship found between insecure attachment styles and trust in CAI in our study. Future studies are suggested to include research-supported methods (eg, recalling relationship experiences and hypothetical scenarios) for activating participants’ attachment systems before conducting the study.

Furthermore, given its sensitive nature, it is also possible that insecure attachment styles affect trust in CAI counseling in a different manner than trust in AI in general. As mentioned in previous sections, we formulated our second hypothesis based on a relevant study conducted by Gillath et al [33]. The AI technologies examined in this study focused on the relationship between attachment insecurity and perceived trust that were designed for more general purposes, such as self-driving vehicles, medical diagnostic apps, and matchmaking services. Unlike self-driving vehicles or matchmaking services, mental health support requires a higher level of empathy and emotional attunement, areas in which AI technology is more likely to be considered to fall short. Research examining the relationship between attachment styles and trust in AI used for sensitive purposes, such as conversational AI for mental health, need to be specific to the context for which they are used.

### General Limitations

There are several limitations that should be mentioned in our study. First of all, one potential obstacle his field of study is the lack of uniformity in defining and measuring AI-related trust. Using different scales to assess trust can lead to the capture of distinct facets of trust and, consequently, generate contradictory results.

Previous research [51] has highlighted the presence of 2 essential components within the overarching concept of trust in AI systems, “user trust potential” and “perceived system trustworthiness.” User trust potential typically encompasses the user’s internal factors, such as attachment styles, that influence their trust in AI systems. In contrast, perceived system trustworthiness focuses on external factors, including user experience (eg, efficiency and effectiveness) and perceived technical trustworthiness (eg, accuracy, security, and privacy). The existing measurement tools for trust do not clearly distinguish and separately assess these 2 aspects, which may lead to inaccurate capture of the relationship and misses out on important nuances.

This signals a pressing need for the development and validation of a more consolidated and clearly structured measurement tool for trust in AI. Such an instrument would greatly enhance the field’s ability to comprehensively assess trust in AI systems. Furthermore, an intriguing avenue for future research is the exploration of which facet of trust, whether internal factors or external factors, exerts a more pronounced influence on actual engagement behaviors, specifically in terms of actual usage. This question holds significant potential for shedding light on the nuances of trust in AI systems and informing practical applications.

Second, our dependent variable CAI adoption intention was measured with a single item on an ordinal scale. Single item may lack the sensitivity to detect subtle differences or changes in the outcome variable, potentially missing important variations in the data. In addition, measuring CAI adoption intention continuously would capture gradual changes more efficiently, leading to more precise description relationship between dependent variable and other independent variables. Multi-item scales should be used to measure adoption intention continuously in future research to increase validity and reliability.

Third, our use of a news article as a vignette to illustrate the use of CAI in mental health support may have implied a subtle positive valence. This could stem from the portrayal of a CAI as a tool that is able to assist individuals with mental health issues. However, as far as possible, we adopted a neutral tone to the vignette, and future studies could consider the portrayal of CAI as a mental health tool with successful (positive) or unsuccessful (negative) outcomes for more generalizable effects.

Furthermore, it is worth noting that disorganized attachment was not examined in the current study. As the first study exploring the relationship between attachment styles and CAI adoption for psychological support, we focused on more clearly defined variables—anxious and avoidant attachment styles—to enable more interpretable and consistent initial insights in this novel area of research. Future research can build on this foundation by incorporating additional insecure attachment styles to generate deeper and more nuanced findings that inform CAI design. In addition, our research participants were sourced through a web-based platform with participants from a single country (the United States). Future research incorporating more diverse samples are encouraged to address these limitations and enhance the generalizability of the findings. Also, although we have excluded participants with previous CAI counseling experience and the results still hold true for the subgroup that reported being entirely unfamiliar with CAI counseling, we acknowledge that future studies would benefit from clearly distinguishing between indirect and direct exposures from which participants gain their familiarity when measuring it.

### Conclusion

In conclusion, our study serves as a pioneering effort in the realm of CAI adoption for mental support, being one of the only papers to examine the impact of attachment styles and perceived trust on CAI adoption. Our findings indicate that perceived trust remains a crucial factor influencing adoption intention; individuals with higher perceived trust are more inclined to try CAI therapies when needed. In addition, attachment anxiety, rather than attachment avoidance, is significantly and positively linked to CAI adoption. These results contribute to the current literature as a good first glimpse into human-CAI relationship and inform the future design of CAI systems, particularly in the mental health setting. By understanding how factors such as perceived trust and attachment styles influence CAI adoption, this study underscores the importance of developing tailored, evidence-based strategies to foster user trust and address specific concerns related to mental health applications. Such strategies may potentially help to mitigate potential risks of CAI adoption, such as overreliance or misuse, ensuring that CAI technologies are safely and effectively integrated into mental health care services. Furthermore, these findings highlight the need for continuous evaluation and adaptation of CAI features to better meet the diverse needs of users, ultimately promoting more positive outcomes in mental health support. Future research should build upon these insights to further refine CAI applications, ensuring they are both user-centered and ethically sound, thereby enhancing their potential to provide effective and accessible mental health care solutions.

## Appendix

Multimedia Appendix 1 Online supplementary material.

## Abbreviations

AI: artificial intelligence
CAI: conversational artificial intelligence
IWM: internal working model
LLM: large language model
OR: odds ratio
