# Supplementary material for: Trust, Anxious Attachment, and Conversational AI Adoption Intentions in Digital Counseling: A Preliminary Cross-Sectional Questionnaire Study
Source: JMIR AI. 2025 Apr 22;4:e68960. doi: 10.2196/68960 (PMC12056427; doi:10.2196/68960)
Supplement: Multimedia Appendix 1 [file ai_v4i1e68960_app1.docx]

**Appendix A: Explanation of Differences Between Pre-Registered and Final Hypotheses**

In this study, there are slight but notable differences between the pre-registered hypotheses and those presented in the final paper, please see Table 1 for more details. These modifications were made to improve the clarity and precision of the paper while remaining aligned with the original research objectives.

**Table S1**.Pre-registered Hypotheses and Hypotheses Presented in the Final Honour Dissertation.

| Hypotheses Version | Content |
| --- | --- |
| Pre-registered Hypotheses | Hypothesis 1: Higher levels of attachment insecurity would be associated with lower intentions to use CAI therapy, even when controlling for attitudes, trust in CAI, and previous experience with AI products. |
|  | Hypothesis 2: Trust towards CAI would predict people’s intention of using CAI for counselling, above and beyond attachment styles. |
| Hypotheses in Dissertation | Hypothesis 1: Higher trust in CAI counselling would lead to higher adoption intentions, controlling for general confounding variables such as age and gender. |
|  | Hypothesis 2: Individuals with higher levels of insecure attachment would show lower adoption intentions, controlling for age and gender. |

**Reasons for Changes**

One of the primary changes involved focusing on age and gender, two more consistent demographic controls that are commonly relevant in adoption behaviour studies, as control variables in the final hypotheses rather than a wider range of potential confounding factors such as previous experience with AI products or attitudes towards CAI. This refinement of focus was made to make the analysis cleaner and more interpretable without over-complicating the model with excessive variables.

Moreover, the order of importance between trust in CAI and attachment styles was reversed in the final hypotheses. This change reflects insights gained during the literature review process. Preliminary evidence indicated that the relationship between trust and adoption intention was more robust and consistent than the link between insecure attachment and adoption intention. This suggests that trust in CAI technology may serve as a more direct predictor of adoption intentions than initially hypothesized. While insecure attachment remains relevant, it was repositioned in Hypothesis 2 to better reflect its secondary role in influencing adoption decisions. Additionally, when examining the effect of trust on adoption intention, the influence of insecure attachment was not controlled for as initially pre-registered. Due to insufficient evidence to confidently underpin the directions between trust, insecure attachment, and adoption intention, we believe it is necessary to streamlin the hypothesis for a simpler but clearer initial examination of these variables.

**Consistency with Original Intent**

While the final hypotheses reflect refinements in terms of variable selection and the order of predictors, they are consistent with the original research intent: exploring the relationship between attachment styles, trust in CAI, and adoption intentions for CAI counselling through ordinal multiple regression. The adjustments were made to line up with a more comprehensive understanding gained through the on-going process of literature review, particularly after the pre-registration, aiming at improving the clarity, relevance, and robustness of the findings.

**Appendix B: Context for Introducing the Concept of CAI Counselling**

**CAI (Conversational Artificial Intelligence) for Mental Health Support**

Just a year ago, Jordan had fulfilled a dream of owning their own bakery, which specialized in the sort of custom-made ornate wedding cakes often featured in baking show competitions.

But last February, all that fell apart, after a car accident left Jordan hobbled by injury, from head to knee. "I could barely talk, I could barely move," they said.

As darkness and depression engulfed Jordan, help seemed out of reach; they couldn't find an available therapist, nor could they get there without a car, or pay for it. Jordan had no health insurance, after having to shut down their bakery.

Therefore, Jordan’s doctor suggested a mental-health app called Therapy. Its chatbot service is free. The chatbot asks questions like, "How are you feeling?" or "What's bothering you?" The computer then analyzes the words and phrases in the answers to deliver supportive messages, or advice about managing chronic pain, for example, or grief.

That is how Jordan found themself on a new frontier of technology and mental health. Advances in artificial intelligence — such as Chat GPT — are increasingly being looked to as a way to help screen for, or support, people dealing with isolation, or mild depression or anxiety. Different from rule-based chatbots which rely solely on pre-written scripts, chatbots driven by more advanced AI technology are called conversational AI (CAI). With machine learning and natural language processing (NLP), CAI enables a more nuanced and personalized understanding and response to user inputs, which has led to the rapid development of CAI counselling in recent years. Human emotions are tracked, analyzed and responded to in CAI counselling. It tries to monitor a patient's mood, or mimic a human therapist's interactions with a patient. The technology aims to provide accessible support. It's an area garnering lots of interest, in part because of its potential to overcome the common kinds of financial and logistical barriers to mental health care, such as those Jordan faced.

**Appendix C: *P* Values for Non-Significant Results**

**Table S2.** Regression Coefficients for CAI Adoption as a Function of Perceived Trust and Control Variables.

| Predictor Variables | Step 1  *b* |  | Step 2  *b* |  |
| --- | --- | --- | --- | --- |
|  |  | *p* |  | *p* |
| Age | 0.011 | .257 | 0.001 | .920 |
| Gender |  |  |  |  |
| Male-Female | 0.237 | .319 | -0.351 | .189 |
| Other - Female | -0.735 | .297 | -0.489 | .570 |
| Trust in  CAI Counselling |  |  | 2.617 | <.001 |
| R^2^ _McF_ | .005 |  | .286 |  |
| R^2^ _McF_ Change |  |  | .281 |  |

*Note.* N = 239.

**Table S3.** Regression Coefficients for CAI Adoption as a Function of Insecure Attachment and Control Variables

| Predicting Variables | Step 1  *b* |  | Step 2  *b* |  |
| --- | --- | --- | --- | --- |
|  |  | *p* |  | *p* |
| Age | 0.011 | .257 | 0.016 | .123 |
| Gender |  |  |  |  |
| Male-Female | 0.237 | .319 | 0.283 | .238 |
| Other - Female | -0.735 | .297 | -0.642 | .370 |
| Attachment Anxiety |  |  | 0.330 | .035 |
| Attachment Avoidance |  |  | -0.355 | .094 |
| R^2^ _McF_ | .005 |  | .012 |  |
| R^2^ _McF_ Change |  |  | .007 |  |

*Note.* N = 239.
